# Comprehensive characterization of pharmacogenes in a Taiwanese Han population

**DOI:** 10.3389/fgene.2022.948616

**Published:** 2022-08-25

**Authors:** Hsing-Fang Lu, Ting-Yuan Liu, Yu-Pao Chou, Shih-Sheng Chang, Yow-Wen Hsieh, Jan-Gowth Chang, Fuu-Jen Tsai

**Affiliations:** ^1^ Department of Medical Research, China Medical University Hospital, Taichung, Taiwan; ^2^ Center for Precision Medicine, China Medical University Hospital, Taichung, Taiwan; ^3^ Department of Laboratory Medicine, China Medical University Hospital, Taichung, Taiwan; ^4^ Division of Cardiovascular Medicine, China Medical University Hospital, Taichung, Taiwan; ^5^ School of Medicine, College of Medicine, China Medical University, Taichung, Taiwan; ^6^ Department of Pharmacy, China Medical University Hospital, Taichung, Taiwan; ^7^ School of Pharmacy, College of Pharmacy, China Medical University, Taichung, Taiwan; ^8^ Epigenome Research Center, China Medical University Hospital, Taichung, Taiwan; ^9^ School of Chinese Medicine, China Medical University, Taichung, Taiwan; ^10^ Division of Medical Genetics, Children’s Hospital of China Medical University, Taichung, Taiwan; ^11^ Department of Biotechnology and Bioinformatics, Asia University, Taichung, Taiwan

**Keywords:** pharmacogenetics, CYP, HLA, population genetics, SNP microarray

## Abstract

Pharmacogenetic (PGx) testing has not been well adopted in current clinical practice. The phenotypic distribution of clinically relevant pharmacogenes remains to be fully characterized in large population cohorts. In addition, no study has explored actionable PGx alleles in the East Asian population at a large scale. This study comprehensively analyzed 14 actionable pharmacogene diplotypes and phenotypes in 172,854 Taiwanese Han individuals by using their genotype data. Furthermore, we analyzed data from electronic medical records to investigate the effect of the actionable phenotypes on the individuals. The PGx phenotype frequencies were comparable between our cohort and the East Asian population. Overall, 99.9% of the individuals harbored at least one actionable PGx phenotype, and 29% of them have been prescribed a drug to which they may exhibit an atypical response. Our findings can facilitate the clinical application of PGx testing and the optimization of treatment and dosage individually.

## Introduction

Pharmacogenetics (PGx) is the study of how individuals respond differently to drugs based on their genes (Lavertu et al., 2018). Genetic guidance for selecting suitable treatment drugs is a key components of precision medicine, especially for identifying responders and nonresponders to a certain treatment, selecting the appropriate dosage, and decreasing the risk of adverse events. To reduce the barrier to implementing PGx in clinical practice, the Clinical Pharmacogenetics Implementation Consortium (CPIC) has provided updated, evidence-based, and peer-reviewed guidelines on PGx based on published clinical annotation (Relling and Klein, 2011). The US Food and Drug Administration (FDA) has evaluated gene–drug interactions and has presented adequate scientific evidence in the form of three tables of PGx associations. These tables, based on genetic markers, guides health-care providers to implement appropriate measures to ensure the safety and efficacy of prescribed medications (Kim et al., 2021).

To standardize the interpretation of gene–drug associations, methods used to identify pharmacogene haplotypes must be consistent among different laboratories to enable the clinical application of the drug (Tayeh et al., 2022). The “star-allele nomenclature” is used to predict the activity or function of pharmacogenes (Robarge et al., 2007). Several pharmacogene naming tools are used to construct the PGx phenotype by using genome sequencing data according to the standardized nomenclature (Twist et al., 2016; Numanagić et al., 2018; Lee et al., 2019). However, because of the small sample size of sequencing studies, determining the PGx star allele frequency distribution in the general population would be difficult (Mauleekoonphairoj et al., 2020). Compared with whole-genome sequencing (WGS) data, the use of single-nucleotide variation (SNV; formerly single-nucleotide polymorphism) array data is more affordable and suitable for biobank studies. The Pharmacogenomics Clinical Annotation Tool (PharmCAT) and the Population-scale Pharmacogenetic Allele and Phenotype Caller (PGxPOP) can be used to construct the PGx gene star allele and determine the phenotype annotation by incorporating definitions from the CPIC database and using genotyping array data as input (Sangkuhl et al., 2020; McInnes et al., 2021). This method generates consolidated and reproducible results, which can be easily compared to interpret the PGx star allele results.

In 2021, the UK Biobank released the PGx allele and phenotype frequencies for clinically relevant genes in 487,409 individuals (McInnes et al., 2021). Most of the participants harbored active PGx variants that may affect treatment outcomes. Besides, nearly a quarter of individuals have been prescribed a medication when they possessed actionable pharmacogene variants toward the medication. Therefore, characterizing PGx phenotypes in larger populations is crucial to determine treatment outcomes. Moreover, to identify the effect of genetic variations on drug responses, the phenotypic constitution of pharmacogenes must be elucidated.

Wang et al. (2021) reported clinical PGx testing results for a Chinese population and demonstrated the benefit of PGx-guided treatment among certain drugs. The Japan PGx Data Science Consortium (JPDSC) collected the data of approximately 3,000 Japanese individuals and built a genotype database of 2.5 million SNPs (Kamitsuji et al., 2015). However, these studies did not apply entire variants to define a PGx haplotype and construct the comprehensive phenotype frequency of the population. To date, pharmacogenes have not been well characterized in the East Asian population, and systemic research on the PGx allele frequency in Han Chinese populations is still lacking. Therefore, we constructed 14 clinically relevant pharmacogenes from nearly 150,000 Taiwanese individuals. Moreover, we evaluated medication prescription records for each participant to identify any unmet need in PGx testing.

## Materials and methods

### Sample collection

We used participant data collected from the China Medical University Hospital Precision Medicine Project (CMUH, Taiwan) and Taiwan Biobank in this study. The CMUH Biobank contains electronic medical records (EMRs) and blood samples of nearly 175,000 individuals. The genotyping method and imputation procedure were described in detail previously (Liu et al., 2021). Clinical information and baseline characteristics of each individual were determined from the EMRs. Regarding the medication records, we mapped the generic names of 66 gene-related drugs to Anatomical Therapeutic Chemical (ATC) codes. We searched the EMR for the ATC codes of prescriptions used in general practice for the participants and found the prescription records of 39 drugs. For each medication, only the first prescription was considered. The prospective Taiwan Biobank contains the clinical information and genetic data of the general Taiwanese population collected from medical institutions and community health centers in Taiwan, including the WGS and genotyping data (Wei et al., 2021). This study was approved by the China Medical University and Hospital Research Ethics Committee (CMUH110-REC3-225).

### Quality control

PLINK1.9 (Purcell et al., 2007) was used for quality control. We excluded SNVs with a call rate of <0.98, a minor allele frequency (MAF) of <0.01, and a Hardy-Weinberg equilibrium (HWE) value of <1 × 10^−6^. At the individual level, SNVs with a call rate of <0.98, a heterozygosity of ±5 standard deviations (SDs) from the mean, or a kinship of >0.354 were excluded. To determine the population structure, we combined the data of 1,000 genome East Asian population (EAS) with our data and conducted principal component analysis by using PLINK2 (Chang et al., 2015). Principal components that did not fall within the three interquartile ranges of 1,000 genome EAS were defined as outliers and excluded from the subsequent analysis.

### Pharmacogene allele construction

After imputation, the variants with an INFO *R* ^2^ of ≥0.3, a MAF of >0.0001, and a *p* value of HWE >1 × 10^−7^ in the variant call format (VCF) were included in the analysis. The default settings of PharmCAT v1.0.0 (Sangkuhl et al., 2020) and PGxPOP (McInnes et al., 2021) were used to generate PGx phenotypes from the imputed VCF file. GRCh38 was applied as reference. These tools extract gene variants and infer star allele haplotypes for each individual by using the CPIC allele definition. To enhance the computational efficiency, we extracted the specific chromosomes of the PharmCAT target region before applying PharmCAT. A total of 10 genes (*CYP2B6*, *CYP2C19*, *CYP2C9*, *CYP3A5*, *CYP4F2*, *DPYD*, *NUDT15*, *SLCO1B1*, *TPMT*, and *VKORC1*) were included in the analysis. If multiple PGx phenotypes were inferred by PharmCAT for an individual, we recorded the phenotype as “NA.” To compare the PGx gene allele and phenotype results between the SNP array and WGS data from the Taiwan Biobank, we inferred the star allele of the phased WGS data (*n* = 1,462).

### HLA imputation

We used the HLA genotype imputation with attribute bagging (HIBAG) (Zheng et al., 2014) to impute HLA alleles with four-digit resolution. The default settings of HIBAG were used during the analysis. Four hypersensitivity-related HLA genes—*HLA-A*, *HLA-B*, *HLA-DQA1*, and *HLA-DRB1*, were imputed in this study. An imputation posterior probability of >0.5 was considered reliable.

### Allele frequency data from pharmacogenomics knowledge base

The Pharmacogenomics Knowledge Base (PharmGKB) (Whirl-Carrillo et al., 2021) curates information from latest pharmacogenomic studies and annotates the gene–drug associations of variants. Moreover, PharmGKB provides a summary of the allele frequencies of pharmacogenes from previous published studies. We downloaded the diplotype and phenotype frequencies in the East Asian population from the PharmGKB website (https://www.pharmgkb.org/page/pgxGeneRef).

### US Food and Drug Administration guidance

We applied the output from PharmCAT and HIBAG and intersected the data with gene drug lists provided by FDA. A total of 14 genes (*CYP2B6*, *CYP2C19*, *CYP2C9*, *CYP3A5*, *CYP4F2*, *DPYD*, *NUDT15*, *SLCO1B1*, *TPMT*, *VKORC1, HLA-A*, *HLA-B*, *HLA-DQA1*, and *HLA-DRB1*) remained. We conducted the PGx study in accordance with the recommendation of the US FDA. We divided PGx associations into high, moderate, and low on the basis of the solidity of evidence from FDA (https://www.fda.gov/drugs/science-and-research-drugs/table-pharmacogenomic-biomarkers-drug-labeling).

## Results

### Population and concordance check

The Taiwanese Han population mainly comprises East Asians. After the exclusion of outliers from the EAS population, the genotyping array information of 172,854 individuals was included in this study. The average age of the cohort was 49.46 ± 20.96 years, and 54% were women. The Taiwan Biobank contains WGS and SNP array data. We used two pharmacogene phenotype callers named PharmCAT and PGxPOP to estimate the phenotype of each pharmacogene. Among most of the genes, the phenotype was consistent between the two tools, with a concordance of >90% (Supplementary Table S1). The detailed frequencies of the haplotype and phenotype of each gene are presented in Supplementary Tables S2, S3. Compared with the output from PharmCAT, the phenotype frequencies generated by PGxPOP were similar to the results from UKBB EAS. Furthermore, we conducted a concordance check between the WGS and SNP array data for alleles and phenotypes among 1,462 individuals. The concordance rate of most of the genes was approximately 0.9; only the concordance rate of the *SLCO1B1* diplotype from PharmCAT was 0.89 (Table 1). All phenotypes were highly consistent between the SNP array and WGS results.

**TABLE 1:** Concordance evaluation of diplotypes and phenotypes between the TWB whole-genome sequencing and TWB imputed array data.

	PharmCAT		PGxPOP	
	Diplotype	Phenotype	Diplotype	Phenotype
*CYP2B6*	99.30%	99.70%	99.52%	99.52%
*CYP2C19*	100.00%	100.00%	100.00%	100.00%
*CYP2C9*	99.80%	99.80%	99.73%	99.73%
*CYP3A5*	99.80%	99.80%	99.73%	99.73%
*CYP4F2*	99.90%	100.00%	99.93%	99.93%
*NUDT15*	99.50%	99.50%	98.49%	99.18%
*SLCO1B1*	88.60%	99.60%	99.66%	99.86%
*TPMT*	99.90%	99.90%	99.93%	99.93%
*UGT1A1*	99.70%	99.80%	99.86%	99.93%
*VKORC1*	99.90%	99.90%	99.93%	99.93%

TWB, Taiwan Biobank.

### Cytochrome p450 star allele analysis

**FIGURE 1:**
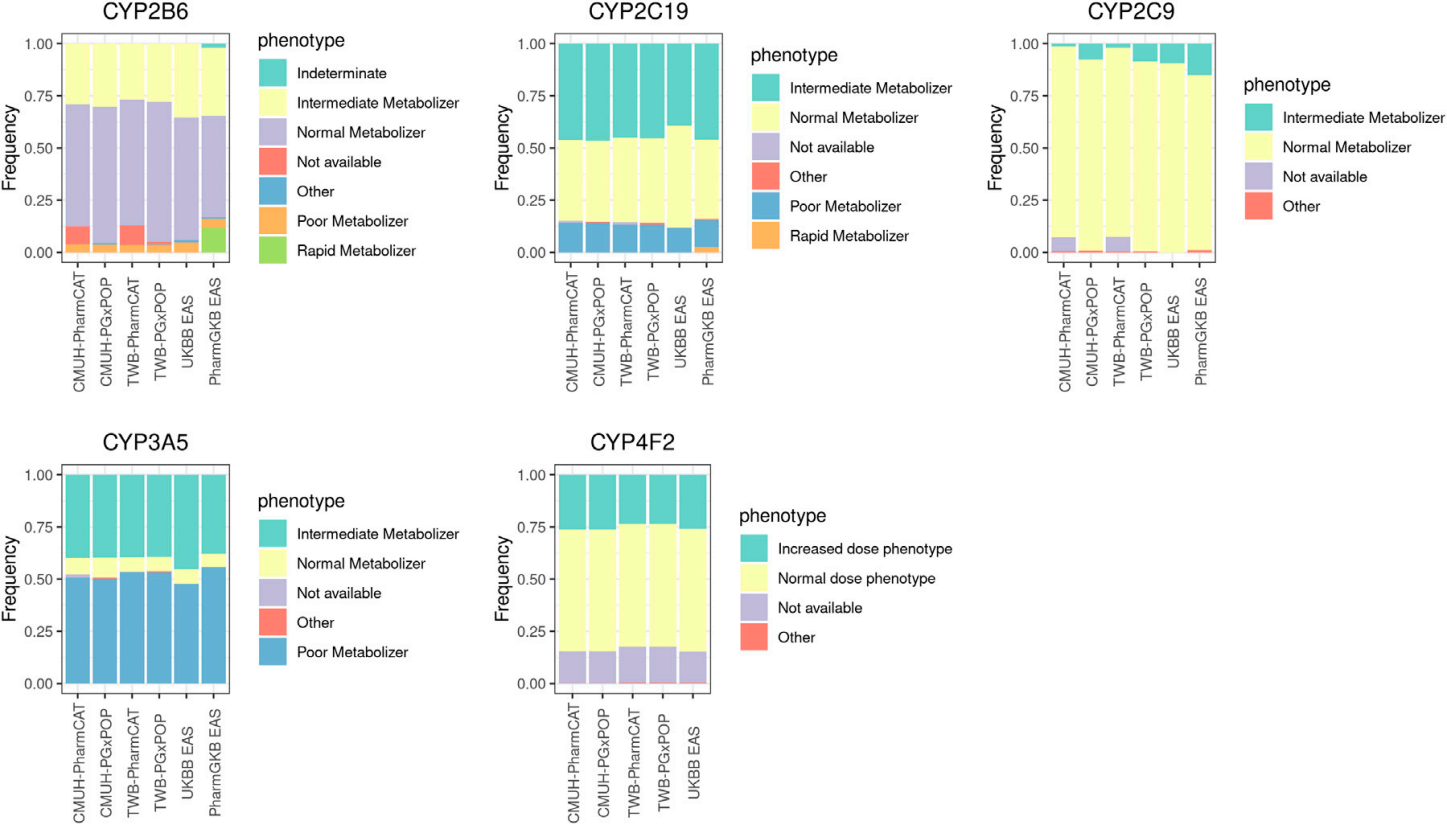
Phenotype frequencies for CYP family genes between different platforms and the East Asian population cohort. The detailed sample size and the frequency of each phenotype are listed in Supplementary Table S3.

The phenotypic distributions of *CYP2B6*, *CYP2C19*, *CYP2C9*, *CYP3A5*, and *CYP4F2* are presented in Figure 1, and the frequency of these genes was compared with that in the East Asian population reported by UKBB and PharmGKB. In general, the phenotype distribution of the four CYP family genes (*CYP2B6*, *CYP2C19*, *CYP2C9*, *CYP3A5,* and *CYP4F2*) was similar between our results and those obtained for the East Asian population. Currently, PharmGKB provides only the allele frequency of *CYP4F2*. The CYP4F2*3 allele frequency was 0.17 in our study and 0.22 in the East Asian population cohort. However, a few individuals in our cohort harbored the CYP4F2*2 allele (0.001).

### Noncytochrome p450 star allele analysis

**FIGURE 2:**
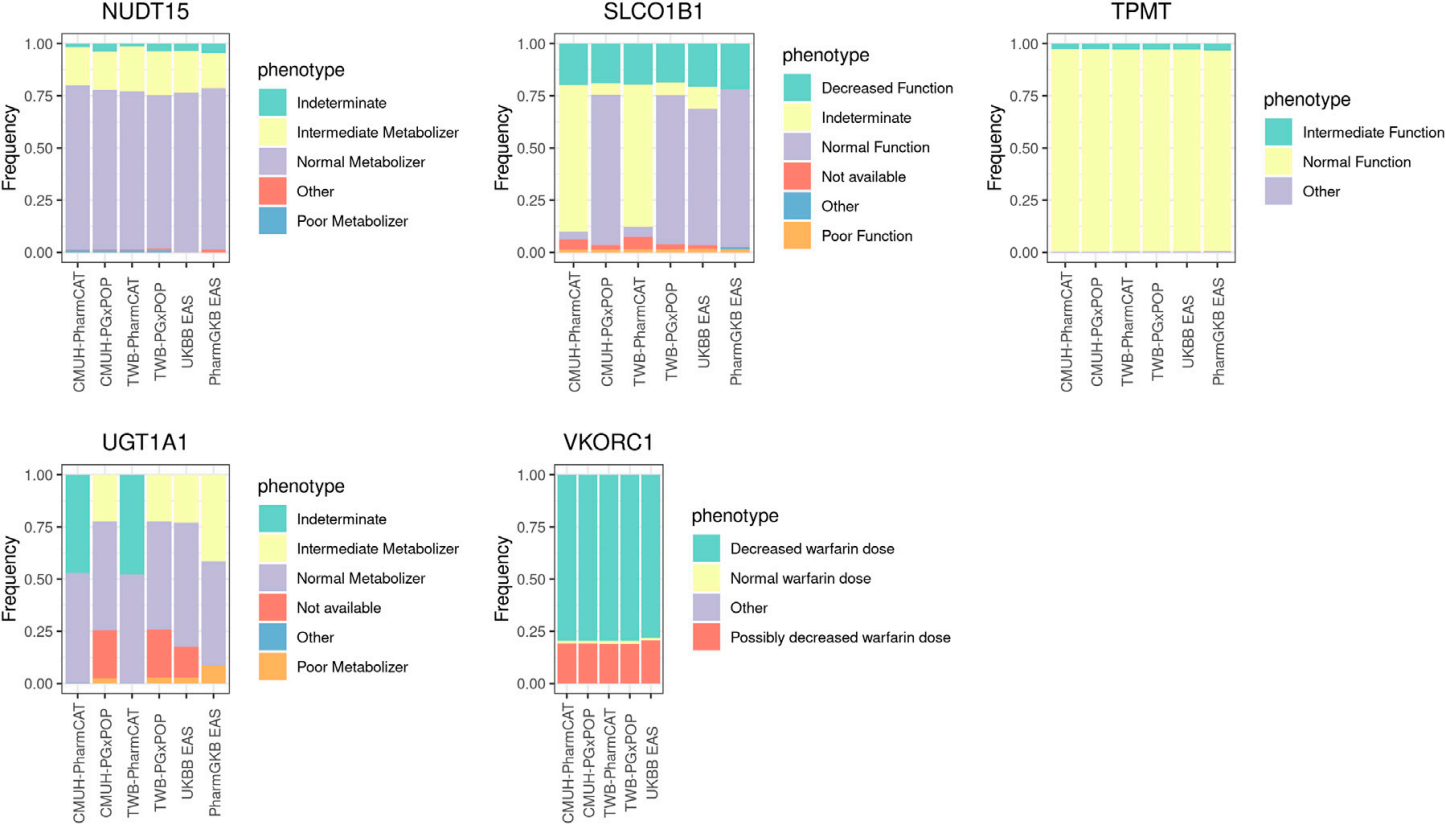
Phenotype frequencies for non-CYP family genes among the genotyping array, WGS data, and East Asian population cohort. The detailed sample size and the frequency of each phenotype are provided in Supplementary Table S3.

Five PGx genes were included (*UGT1A1*, *TPMT*, *NUDT15*, *SLCO1B1*, and *VKORC1*) in our study (Figure 2). In general, the phenotype frequency distributions of *TPMT*, *NUDT15*, and *VKORC1* in our cohort were similar to those in the East Asian population. Some differences were noted among different phenotype callers. For example, the most common phenotype of *SLCO1B1* was indeterminate (70%) from PharmCAT, whereas normal function was the dominant phenotype in PGxPOP and UKBB EAS populations. The most common star allele of *SLCO1B1* was *37 (61%) in the PharmGKB EAS population, while *1B appeared in approximately 60% of the CMUH array and UKBB EAS samples. Moreover, we evaluated the *UGT1A1* haplotype distribution and observed that the *60 allele frequency was 23% in our cohort but the allele did not appear in the PharmGKB EAS (Supplementary Table S2).

Our results revealed that nearly 80% of the *VKORC1* rs9923231 diplotype is TT, which may increase the sensitivity of an individual to warfarin. The allele frequency of the rs9923231 variant T was 0.89 in the CMUH genotyping array and 0.87 in the East Asian population cohort.

### HLA results

**FIGURE 3:**
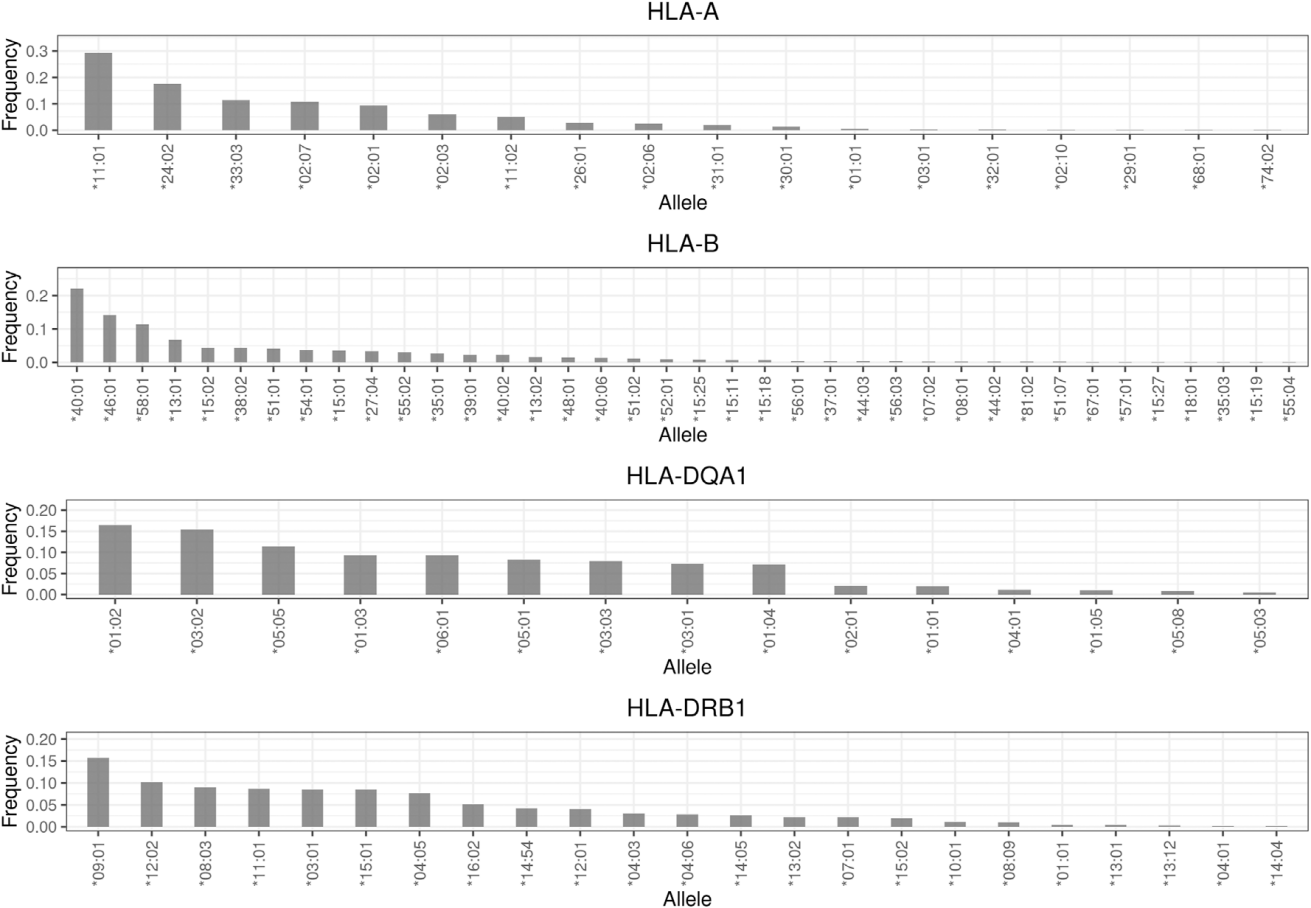
Allele frequency distribution of HLA genes. Frequencies less than 0.001 are not shown in this plot.

The detailed frequency distributions of the drug responses of four HLA genes, namely *HLA-A*, *HLA-B*, *HLA-DRB1*, and *HLA-DQA1*, are described in Supplementary Tables S3–S6. In brief, the most common alleles of these four genes were HLA-A*11:01 (29.3%), HLA-B*40:01 (22%), *HLA-DQA*1*01:02 (16.5%), and *HLA-DRB*1*09:01 (15.7%), respectively (Figure 3). Among the drug hypersensitivity–related alleles, HLA-B*58:01 (11.4%) was the most common, followed by HLA-B*15:02 (4.4%). The frequency of the other three alleles (HLA-A*31:01, HLA-DQA1*02:01, and HLA-DRB1*07:01) was approximately 2%. The least common allele was HLA-B*57:01, with a frequency of 0.2%. In general, the frequencies of the four *HLA* alleles were consistent with those reported in a previous study (Huang et al., 2020).

### Medication use

**FIGURE 4:**
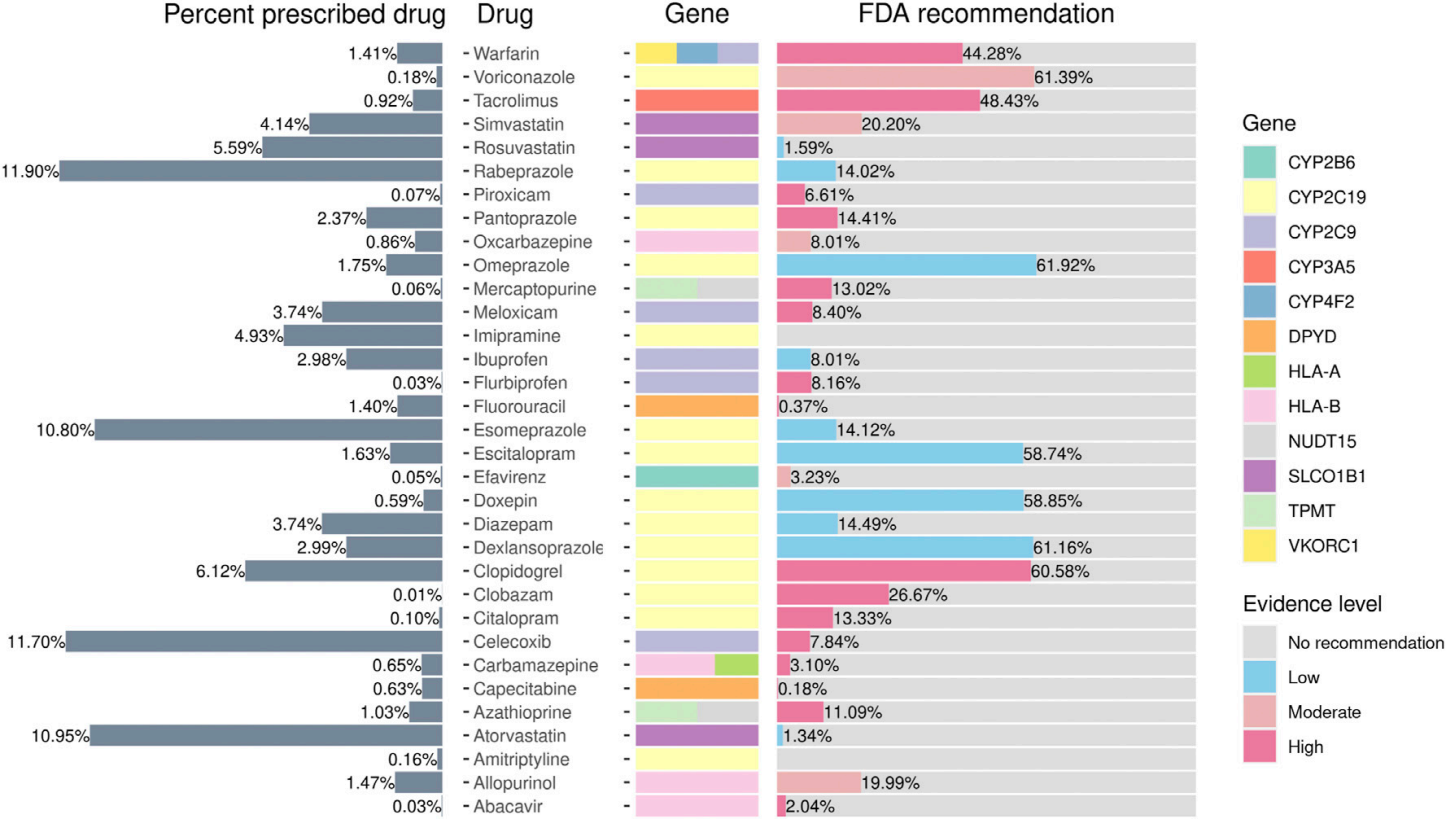
Prescribed percentages of pharmacogene-related drugs according to FDA guidelines. The figure presents the phenotype frequencies from the PGxPOP of the subjects from the CMUH that have ever been prescribed the treatment, the generic name of the drug, the genes that affect the treatment, and the percentage of the FDA guidance evidence level from left to right.

We extracted data on the prescription rates of 12 medications relevant to PGx-related treatment from the CMUH EMR. We observed that 99.9% of individuals may atypically respond to at least one medicine. Furthermore, 29% of the individuals harbored the actionable variants for the prescribed drug. As presented in Figure 4, the top three drugs prescribed were the protein pump inhibitor rabeprazole (11.9%), NSAID celecoxib (11.70%), and HMG-CoA reductase inhibitor atorvastatin (10.95%). Notably, 6,414 (61%) clopidogrel recipients belonged to the phenotype that warrants a change in treatment according to the US FDA’s recommendation. Among them, 46% were the intermediate metabolizers and 14% were the poor metabolizers of *CYP2C19*. The anticoagulant warfarin is another drug that merits investigation. Although only approximately 1.41% of the individuals received warfarin in the present cohort, 44% of them may require the adjustment of the treatment dosage. Moreover, the dosage may need to be modified in 48% of tacrolimus users (*n* = 772) to prevent rejection risk.

## Discussion

To date, this is the largest PGx study to report the distribution of diplotypes and phenotypes in the Taiwanese population. This study comprehensively investigated the haplotype frequency of 14 treatment response–related genes. Moreover, through the incorporation of prescription records in the analysis, the potentially affected population of the atypical PGx star allele on drug responses was investigated.

Although the call rates of the alleles were not identical between the genotyping array and WGS data, most of the identified phenotypes were consistent. Because one phenotype may contain different alleles, the response to a medication is mainly driven by the phenotype rather than the allele. Moreover, the phenotypic distribution of most pharmacogenes is consistent with that of the East Asian population. These results indicated that imputed genotyping data may generate robust phenotypes for each individual, which may help in treatment selection in clinical settings.

The antiplatelet agent clopidogrel is widely used to reduce the risk of cardiovascular events in high-risk patients (Yusuf et al., 2001). *CYP2C19* plays a crucial role in the bioactivation of clopidogrel (Sangkuhl et al., 2010). Exposure to the active clopidogrel metabolite was significantly lower in the *CYP2C19* intermediate metabolizers and poor metabolizers, thereby diminishing the antiplatelet effect (Brandt et al., 2007; Varenhorst et al., 2009; Duarte and Cavallari, 2021). Moreover, real-world evidence indicates that the atherothrombotic risk was lower in the carriers of the *CYP2C19* loss-of-function variant treated with alternative drugs than in those receiving clopidogrel (Beitelshees et al., 2022). Our study demonstrated that 61% of the clopidogrel recipients exhibited the phenotype that may not have had sufficient exposure to the active clopidogrel metabolite. These results indicate that phenotype-guided treatment is crucial in clinical practice and help in prescribing suitable drugs to patients.

The haplotype and phenotype frequencies of *UGT1A1* and *SLCO1B1* were not consistent with those in the East Asian population from PharmGKB, which can be explained by several plausible reasons. First, rare variants and structural variations play a crucial role in star allele haplotyping. However, the SNV microarray may not fully capture these variations. Second, frequency distributions in the East Asian population of the PharmGKB were determined in the basis of previous studies. Because these studies did not test for all known variant alleles (Premawardhena et al., 2003; Teh et al., 2012), once the alleles were negative for sequenced variation, the star allele may default assign to *1. Moreover, these studies had small sample sizes (Maeda et al., 2014) and focused on specific phenotypes; therefore, the results may not be representative of the general population. Third, a previous study demonstrated that haplotype call rates are affected by the design of pharmacogenomic assays, and assays that examine various variants generate more accurate results compared with those that examine a relatively limited number of variants (Samwald et al., 2015). Therefore, the discrepancies in the phenotype frequency may be caused by the differing variant coverage and design of each platform (Kalman et al., 2016). Additional large-scale population-based studies should be conducted to comprehensively determine the frequency of pharmacogenes through the incorporation of structural variation information.

*CYP2D6* is an important pharmacogene involved in the metabolism of nearly 20% of widely used medications (Taylor et al., 2020). In addition to SNVs, the precise haplotype definition of *CYP2D6* depends on copy number variants and structural alterations (Beoris et al., 2016). A previous study indicated that genotyping arrays cannot capture variations in *CYP2D6* comprehensively due to the highly polymorphic nature of this gene (Twesigomwe et al., 2020). The phenotype of *CYP2D6* called by the genotyping array may be biased consequently (McInnes et al., 2021). Therefore, we did not include the phenotype frequency of *CYP2D6* in this study.

In conclusion, our study comprehensively evaluated the allele and phenotypic distributions of clinically crucial PGx genes in a Taiwanese Han population through systemic imputation and annotation. By analyzing the prescription information from the EMRs, we identified the effect of each drug on a specific population and highlighted the importance of PGx in clinical settings. The study results provide insights into the phenotypic frequencies of clinically relevant pharmacogenes and may be used as reference for designing future PGx studies.

## Data Availability

The original contributions presented in the study are included in the article/Supplementary Materials, further inquiries can be directed to the corresponding authors.
